# Radiation Oncology Follow-Up of Prostate Cancer Survivors Following Completion of Radiotherapy: A Population-Based Study

**DOI:** 10.3390/curroncol33010049

**Published:** 2026-01-15

**Authors:** Joshua O. Cerasuolo, Jonathan Sussman, Ian S. Dayes, Rinku Sutradhar, Manaf Zargoush, Hsien Seow

**Affiliations:** 1 Department of Health Research Methods, Evidence, and Impact, Faculty of Health Sciences, McMaster University, Hamilton, ON L8N 3Z5, Canada; 2 ICES McMaster, Faculty of Health Sciences, McMaster University, Hamilton, ON L8S 4L8, Canada; 3 ICES North, Health Sciences North Research Institute, Sudbury, ON P3E 2H2, Canada; 4 Medical Sciences Division, NOSM University, Sudbury, ON P3E 2C6, Canada; 5 Department of Oncology, Faculty of Health Sciences, McMaster University, Hamilton, ON L8V 5C2, Canada; 6 ICES, Toronto, ON M4N 3M5, Canada; 7 Dalla Lana School of Public Health, University of Toronto, Toronto, ON M5T 3M7, Canada; 8 Institute of Health Policy, Management, and Evaluation, University of Toronto, Toronto, ON M5T 3M6, Canada; 9 Health Policy and Management Area, DeGroote School of Business, McMaster University, Hamilton, ON L8S 4M4, Canada

**Keywords:** prostate cancer, survivorship care, retrospective cohort, population-based, Gleason

## Abstract

Prostate cancer survivors require coordinated long-term care after treatment. We examined patterns of follow-up care and identified characteristics associated with the frequency of radiation oncology (RO) visits during survivorship. We conducted a population-based cohort study of men diagnosed with prostate cancer between April 2010 and March 2019 in Ontario, Canada, who underwent first-line radiotherapy. Survivorship began three years following radiation. Using a recurrent event framework, we identified demographic and clinical characteristics associated with the rate of RO follow-up. Survivors seeking RO follow-up declined by 46.2% over five years of survivorship. Higher-risk characteristics, such as higher ISUP grade, higher stage, detectable prostate-specific antigen (PSA) score, and receipt of brachytherapy and/or hormones, were associated with more frequent RO visits. For instance, relative to International Society of Urological Pathology (ISUP) Grade 1, those with Grades 3 through 5 experienced follow-up rates that were 20%, 25%, and 34% higher, respectively. Despite concordance between patient risk and rate of RO follow-up, 23.6% of survivors continued to visit their RO providers into their fifth year of survivorship, more than half of whom were ISUP grades 1–2. Primary care follow-up remained stable. While frequency of RO follow-up appropriately reflected patient risk profile, many low-risk survivors still sought long-term RO-led care. This suggests an opportunity to encourage lower-risk prostate cancer survivors to seek follow-up care with their general practitioner.

## Simple Summary

Prostate cancer survivors often need long-term follow-up to manage side effects of treatment and monitor relapse or progression. It is unknown which patients seek routine follow-up visits, and how often, at specialized cancer hospitals. In this study, we looked at which patient characteristics were related to how frequently survivors saw their radiation oncologist, starting three years after completing radiation treatment. We found that patients at higher risk had more frequent follow-up visits with radiation oncology, while lower-risk patients were seen less. Overall, the number of survivors returning for radiation oncology follow-up dropped by more than 46% over five years, which suggests that care is being tapered appropriately over time. Still, more than 23% of survivors were seeing their radiation oncologist in the fifth year after treatment, and over half of these patients were considered lower risk. This shows that even though follow-up generally aligned with patients’ needs, many lower-risk survivors continued longer-term follow-up with radiation oncology. Transitioning the ongoing care of lower-risk patients to their primary care providers enables long-term follow-up closer to home, while ensuring a sustainable cancer care system.

## 1. Background

Prostate cancer is the most common malignancy among Canadian men, accounting for 20% of all new diagnoses, and has a favourable 5-year survival rate of 91% [1]. The growing population of prostate cancer survivors underscores the importance of survivorship as a crucial area of research [2,3,4]. Survivorship refers to the phase of cancer care following initial diagnosis and treatment [5,6,7,8]. This stage focuses on addressing the long-term physical, emotional, and psychosocial needs of cancer survivors as they transition away from active treatment and adapt to living with lasting effects of the disease [9,10,11]. For prostate cancer, this includes the coordination of care among oncology, urology, and primary care across the patient’s lifespan, culminating in the easing of oncology-led care after the completion of post-treatment follow-up [12,13]. Prostate cancer survivors often face challenges in navigating a complex healthcare system, as they seek the necessary long-term care and support particularly during the transition away from oncology-led care within a specialized cancer institution [14,15,16]. Transitioning to general practitioner (GP)-led care in their home community can be difficult, as survivors adjust to being managed by a primary care provider outside of the auspices of the cancer care system, while concomitantly identifying and addressing treatment-related side effects and adjusting to new surveillance expectations [17]. Despite these challenges, transitioning away from specialized oncology care is an important and necessary step in the care continuum to avoid placing undue constraints on an already overburdened cancer care system. Real-world evidence illustrating patterns of follow-up care as well as identifying demographic and clinical characteristics associated with RO involvement among prostate cancer survivors are limited. We aimed to characterize survivorship care following completion of first-line radiotherapy in a population-based cohort, focusing on patterns of RO follow-up and factors associated with the presence and frequency of RO visits. Our findings can inform the development of personalized, risk-specific prostate cancer survivorship guidelines to facilitate a safe and efficient transition from the cancer care system, ultimately improving the long-term health outcomes, well-being, and quality-of-life of patients as they navigate the healthcare system during survivorship.

## 2. Methods

### 2.1. Study Design and Data Sources

Using the Ontario Cancer Registry (OCR), we conducted a population-based retrospective cohort study of prostate cancer survivors who opted for radiation as their first-line therapy. The OCR captures over 95% of all incident cancers diagnosed in Ontario, Canada [18,19], and comprises detailed clinical information such as site, morphology, TNM (tumor, node, metastasis) staging, International Society of Urological Pathology (ISUP) grade via Gleason score, and diagnosis date. We deterministically linked the OCR to other administrative healthcare databases to comprehensively examine all health services and treatments sought after diagnosis. Data regarding receipt of cancer treatment(s) were obtained from the Cancer Activity Level Reporting database, New Drug Funding Program database, Ontario Drug Benefit Program database, and cancer clinic data collected via the National Ambulatory Care Reporting System. Office-based outpatient visits to radiation oncology, urology, and primary care were ascertained from physician remuneration data in the Ontario Health Insurance Plan database; the ICES Physician Database (IPDB), via encrypted physician identifiers, discerned medical specialty. Demographic and mortality data were extracted from the Registered Persons Database. Lastly, laboratory tests for prostate-specific antigen (PSA) were captured in the Ontario Laboratory Information System. Detailed descriptions of all data holdings and definitions used in this study are provided in the Supplemental Material (Tables S1 and S2, respectively, online only). These databases were linked and analyzed at ICES (formerly known as the Institute for Clinical Evaluative Sciences) using a unique encoded patient identifier.

### 2.2. Cohort Description

Our cohort included all Ontario residents diagnosed with incident prostate cancer between 1 April 2010, and 31 March 2019, inclusive (Figure S1, online only). Prostate cancer diagnoses were extracted from the Ontario Cancer Registry (OCR) using the International Classification of Disease, Oncology, 3rd Revision (ICD-O-3: C61). Given that our focus was to study those who underwent radiation as their first-line therapy (i.e., not salvage radiation), we excluded patients with prostate cancer who did not initiate radiation within one year of their diagnosis, received radical prostatectomy, or whose first-line radiotherapy regimen duration was atypical, defined as exceeding 90 days. We also excluded patients not suitable for survivorship; this excluded anyone who received radiation to a body region other than the prostate or pelvis (e.g., lung, brain), received chemotherapy, or was diagnosed with de novo metastatic disease. Additionally, we excluded in situ diagnoses, those who died before the start of survivorship, those aged younger than 18 years or older than 105 years, non-residents of Ontario, those not eligible for Ontario’s universal provincial health coverage (i.e., emigration), or non-community dwellers (e.g., living in a long-term care facility or receiving palliative care services). The creation of our incident survivorship cohort resembled that of previous studies examining survivorship outcomes of common cancers [20,21,22,23].

**Figure 1:**
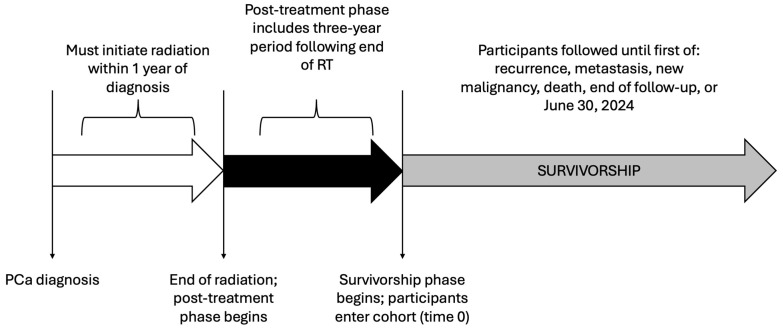
Study participant timeline.

Patients eligible to enter the cohort were observed during two distinct and consecutive phases of care: (1) post-treatment and (2) survivorship (Figure 1). The post-treatment phase of care encompassed the three-year period immediately following the completion of first-line radiotherapy, as three years was an adequate duration for the patient to clinically stabilize. This includes completion of hormonal therapy and related follow-up [24], and for the PSA to reach nadir [25]. This approach is further supported by survey evidence indicating that Canadian primary care practitioners report comfort assuming responsibility for survivorship care around this time frame [26]. To minimize misclassification of patients entering survivorship, we implemented a “wash-out” to exclude individuals likely to represent (1) early biochemical relapse prior to survivorship or (2) higher-risk cases intended to remain on prolonged hormonal therapy. Specifically, we removed patients who dispensed hormones during the 6-month interval immediately before the start of survivorship (i.e., 2.5–3 years after radiotherapy). We evaluated the robustness of our findings through two sensitivity analyses: (1) applying a longer 1-year wash-out period and (2) no wash-out.

During the survivorship phase of care, we observed all outpatient follow-up visits to RO, urology, and primary care. In Ontario, medical oncology is not involved in routine post-radiotherapy follow-up care for prostate cancer and was not included in this study. Participants were followed throughout survivorship until re-initiation of radiation, receipt of chemotherapy, or dispensation of hormonal therapy (i.e., probable recurrence/relapse and/or metastases), diagnosis of a new primary malignancy, death, loss to follow-up, or 30 June 2024, whichever occurred first.

### 2.3. Clinical Endpoints

Our primary outcome was the rate of RO follow-up during the complete survivorship phase of care. Secondarily, we examined three mutually exclusive groups based on the frequency of follow-up during the first year of survivorship [i.e., days 1096–1460 (4th year) following the end of radiation]: (1) those who ‘transitioned’ in the first year of survivorship (i.e., no outpatient visits with a RO), (2) those who received ‘de-intensified’ care (i.e., one or two outpatient visits with a RO), and (3) those who received active follow-up (i.e., three or more RO visits). RO administers treatment and follow-up care within highly specialized cancer hospitals. The absence of such visits during the first year of survivorship identifies patients who have left the cancer care system to seek continuing care and ongoing surveillance with their GP [20].

### 2.4. Statistical Analysis

Baseline characteristics were compared across those who transitioned, received de-intensified follow-up, or received active follow-up (Table 1). Standardized differences (SD) > 0.1 were considered statistically important, consistent with large-sample epidemiological studies [27]. We employed a recurrent event framework to identify demographic and clinical characteristics associated with the rate of RO follow-up during survivorship (primary outcome). Using an Anderson-Gill counting process model (Table 2; Model 1), we regressed baseline characteristics on RO follow-up visits that occurred during survivorship until the patient was censored.

Over 30% of our cohort transitioned in the first year of survivorship (i.e., no RO visits). Therefore, we employed log-binomial regression to estimate adjusted risk ratios (aRRs) by modelling the probability of at least one outpatient RO visit during the first year of survivorship against baseline characteristics (Table 2; Model 2) [28]. Covariates for both models were selected based on clinical domain knowledge of the authors, conditional on data availability. This included age at diagnosis, geographic remoteness [29], socioeconomic status [30,31,32], access to primary care, and number of comorbidities quantified via the CIHI Population Grouping methodology [33]. We also included clinical information such as diagnosis of a previous malignancy, ISUP grade, TNM stage, most recent PSA score prior to commencement of survivorship, receipt of brachytherapy and/or hormones, and the occurrence of radiation-related complications. Gleason scores less than or equal to 6 were categorized as ISUP Grade 1, 7 (3 + 4) as Grade 2, 7 (4 + 3) as Grade 3, 8 as Grade 4, and 9–10 as Grade 5. Continuous covariates not linearly associated with the outcome (i.e., age and remoteness) were accounted for using restricted cubic splines. Furthermore, since the Anderson-Gill model can accommodate time-varying covariates, we included the number of follow-up visits to urology or primary care during survivorship.

All statistical analyses were conducted using SAS Enterprise Guide version 8.3 (Cary, NC, USA). This study received ethics approval from the Hamilton Integrated Research Ethics Board (project #15712).

## 3. Results

Our cohort included 12,866 Ontario men diagnosed with prostate cancer between April 2010 and March 2019 who received first-line radiotherapy. The mean age at diagnosis was 73.5 years (standard deviation [SD] = 7.3), ranging from 46 to 95 years, and survivors were followed for an average of 1769 days (SD = 1012.0) during the survivorship period. Over the full observation window (maximum 4012 days), 15.5% (n = 1994) experienced a suspected recurrence or metastasis, defined as re-initiation of radiation, receipt of chemotherapy, or dispensation of hormones, 5.4% (n = 688) developed a new primary cancer, and 11.9% (n = 1525) died. The remaining participants (n = 8659) were lost to follow-up (i.e., emigration) or censored at the end of follow-up. Kaplan-Meier curves for all-cause mortality are presented in Figure S2.

**Figure 2:**
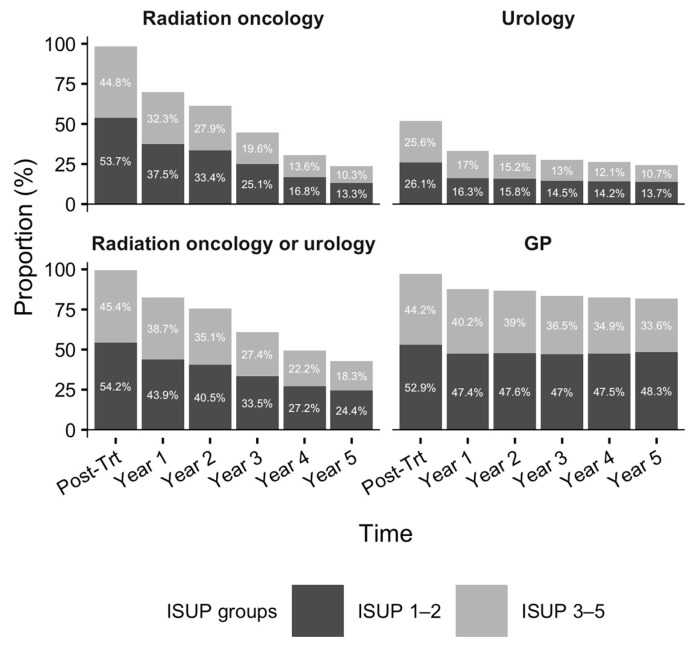
Proportion of patients with at least one office visit to radiation oncology, urology, or general practitioner during the first five years of survivorship. Footnote: Denominators for the post-treatment phase to the 5th year of survivorship (in order) are n = 12,866, n = 12,866, n = 12,145, n = 11,104, n = 8836, and n = 6887, respectively. Figure 2 was produced using R version 4.5.1 [34].

During the post-treatment phase, nearly all survivors had at least one RO (98.5%, n = 12,671) and GP (97.0%, n = 12,485) visit, while 51.7% (n = 6657) saw a urologist (Figure 2). As survivorship progressed, the proportion seeing RO providers declined by 46.2%, whereas decreases in urology (8.9%) and primary care (5.7%) visits were modest. By the fifth year of survivorship, 23.6% continued to receive RO follow-up; however, more than 50% of these were low-risk survivors (ISUP Grades 1–2). Moreover, 24.4% and 81.9% sought care from a urologist and GP, respectively.

Baseline characteristics were compared by the frequency of care sought by radiation oncology during the first year of survivorship (i.e., days 1096–1460 following the end of radiation) (Table 1). In the first year, 30.2% (n = 3884) of study participants were considered as ‘transitioned’, in other words, had no follow-up visits with RO. Nearly two-thirds (64.7%; n = 8321) sought ‘de-intensified’ care within a cancer center (i.e., 1–2 visits with RO). A small proportion of the patients (5.1%; n = 661) were actively receiving RO-led care (i.e., three or more visits). Clinical characteristics, including TNM stage, ISUP grade, and most recent PSA score, were similar between patients who transitioned and those who sought ‘de-intensified’ RO follow-up. However, at baseline, among those who sought active care (i.e., 3+ RO visits) relative to those who transitioned, there were 3.7% more patients with ISUP Grade 5, 7.2% more with ISUP Grade 3, and 11.7% fewer patients with low-grade cancer (ISUP Grades 1 or 2). Similarly, 13.0% of survivors under active care were diagnosed with TNM stage III versus 7.8% among those who transitioned. Likewise, a detectable PSA score was 20.7% more prevalent among those under active RO-led care. More survivors who sought de-intensified or active RO follow-up received a combined administration of external beam radiation and brachytherapy (18.9% and 18.0%, respectively), while conversely, external beam radiation directed only to the prostate was most common among transitioned survivors (65.4%).

The volume of outpatient radiation oncology (RO) follow-up throughout the survivorship phase of care was largely driven by clinical characteristics of the cohort-qualifying prostate cancer (Table 2). Upon multivariable adjustment, we discovered that ISUP grade, TNM stage, and most recent PSA score exhibited a dose–response relationship with the frequency of RO follow-up: compared to patients with ISUP Grade 1, RO follow-up rate increased for Grades 3, 4, and 5 by 20%, 25%, and 34%, respectively. Alike, stages II and III had a higher rate of follow-up than stage I (15% and 29%, respectively), as well as those with a detectable PSA score relative to those with an undetectable score (21%). Receipt of brachytherapy, whether as a monotherapy or combined with external beam radiation, or hormonal therapy was also associated with a higher rate of radiation oncology follow-up (16%, 11%, and 5%, respectively). As the number of pre-existing comorbidities increased, so too did the rate of RO follow-up (5% per 3 additional comorbidities). The occurrence of urological and rectal/anal complications attributed to radiotherapy also increased the rate of follow-up by 20% and 10%, respectively, as well as those requiring open surgery (25%). Lastly, the involvement of other medical specialties relevant to the ongoing care of prostate cancer survivors—urology and family medicine—influenced the frequency of RO-led care. For instance, each additional outpatient visit to urology or family medicine during survivorship reduced the rate of RO follow-up by 12% and 5%, respectively. Additionally, we discovered that patients not rostered to primary care experienced an 18% lower rate of radiation oncology follow-up.

Finally, like the rate of radiation oncology follow-up, we demonstrated that the probability of seeking any RO care in the first year of survivorship (i.e., at least 1 visit) was associated with clinical risk and complexity, reflecting survivors who are not suitable for transition away from radiation oncology and require more intense monitoring within the cancer care system. This included those with a detectable PSA score, and receipt of brachytherapy and/or hormones. Conversely, patients of advanced age, those residing in more rural and remote regions, and those not rostered to a primary care physician were more likely to transition out of the cancer care system. ISUP grade or TNM stage were not related to the probability of any RO follow-up during the first year of survivorship.

## 4. Discussion

In this population-based study of prostate cancer survivors treated with first-line radiotherapy, we characterized patterns of follow-up across radiation oncology, urology, and primary care, and identified factors associated with more frequent radiation oncology (RO) involvement during survivorship. More than 30% of survivors transitioned away from RO-led care during the first year of survivorship, while others continued to receive RO follow-up later into their survivorship phase of care. Overall, our findings suggest that radiation oncology largely follow a risk-informed approach, as survivors with higher TNM stage, detectable PSA, higher ISUP grade, radiation-related complications, or those receiving brachytherapy and/or hormonal therapy were seen more frequently by RO providers. These patterns are consistent with the clinical need for closer monitoring among survivors at higher risk of recurrence or with more complex post-treatment trajectories, and equally, are reflective of lower-risk survivors who are better positioned to completely transition out of the cancer care system and shift toward long-term GP-led surveillance.

Despite the concordance between risk features (e.g., ISUP grade) and frequency of RO follow-up during survivorship, a considerable proportion of lower-risk survivors—those with ISUP grades 1 or 2—continued to receive RO follow-up in the fifth survivorship year, or eighth year following completion of radiation. While some of these visits may reflect lingering long-term treatment-related side effects, patient preference, or limited access to a primary care physician, these findings highlight an opportunity to optimize survivorship pathways by identifying appropriate candidates for earlier transition to general practitioner (GP)-led care. The inverse association between RO follow-up frequency and concurrent outpatient visits to urology and primary care further suggests that coordination with other relevant specialties may help facilitate transition away from RO-led care. Key tenets of cancer survivorship include the safe transition of patients from oncological to primary care [35], in addition to effective coordination and communication across medical specialties [36] (oncology, urology, and primary care) to maintain care continuity and optimize long-term outcomes and quality of life for survivors [37,38,39,40]. As over one-fifth of study participants, followed for an average of five years, experienced probable recurrence, metastasis, or a new malignancy, this emphasizes the importance of ongoing follow-up and vigilant monitoring for higher-risk patients, to ensure timely identification of disease progression, complications of treatment, or new health issues [41,42].

The lack of association between ISUP grade and the probability of any RO visit in the first year of survivorship warrants discussion. This does not imply that RO disregards clinical grade; instead, it likely reflects the multi-factorial influence on early survivorship follow-up beyond ISUP grade alone, including PSA kinetics, treatment modality, and patient preference. The noted dose–response relationship between ISUP grade and overall rate of RO follow-up supports our assertion that RO appropriately maintains a higher frequency of follow-up for survivors with higher-grade disease, even if the specific timing of transition away from the cancer care system may be influenced by additional considerations beyond clinical risk. However, baseline characteristics were more similar between patients who received “de-intensified” follow-up care, compared to those who transitioned to GP-led care (no RO follow-up), suggesting there still may be survivors receiving ‘de-intensified’ care who are suitable candidates to transition exclusively to GP-led care during survivorship.

Strengths of our study included the use of a population-based cohort of prostate cancer survivors in Ontario, Canada spanning a decade, which included unique patient identifiers to facilitate data linkage across all healthcare sectors to elicit a complete picture of their care pathway. Additionally, we included key clinical indicators relevant to prostate cancer, including ISUP Grade, TNM stage, and PSA score. However, utilizing physician claims data limited our ability to definitively determine whether follow-up visits reflected routine survivorship care versus occurrence of cancer recurrence or metastasis, a new primary malignancy, or management of worsening symptoms and/or radiation-related toxicity. To mitigate misclassification of visits related to disease progression or active cancer care, we censored observation of participants who re-initiated radiation, dispensed hormones, received chemotherapy, or were diagnosed with a new primary malignancy. Furthermore, we included radiation-related complications as a covariate in our analysis to account for its role in radiation oncology follow-up throughout survivorship.

Our use of administrative healthcare databases limits our ability to account for other factors that could influence decision-making, such as patient and/or physician preferences, or financial, medical, or logistical factors [43,44,45,46,47]. In addition, because this study was conducted within Canada’s single-payer healthcare system, follow-up patterns may differ in jurisdictions with different access, care, and reimbursement models, which may hinder external validity. Finally, our study exclusively examined patients diagnosed with prostate cancer who underwent first-line radiation as a monotherapy and may not be generalizable to survivors treated with prostatectomy alone, prostatectomy followed by salvage radiation, or non-definitive management such as active surveillance or watchful waiting [48]. Radiation offers a non-surgical alternative for eligible patients [48] but entails a trade-off between clinical benefit [49] and post-radiotherapy complications [50] and may partly explain why some lower-risk survivors continue specialist follow-up longer than expected. Future work should explore survivorship care pathways of other primary modalities. Further, we suggest quantifying the real-world clinical effectiveness of transitioning (e.g., comparing risk of cancer-related adverse events among those under oncology- versus GP-led care), which in absence of a superior alternative, may promote GP-led care, especially among low-risk survivors.

## 5. Conclusions

In summary, our results suggest that higher-risk prostate cancer survivors are monitored more frequently by RO providers, consistent with clinical need. However, many lower-risk survivors continue to receive RO-led follow-up several years after completing treatment, indicating an opportunity to build risk-stratified survivorship pathways that support safe transitions to GP-led care. Such efforts may help alleviate pressure on cancer centres while ensuring appropriate monitoring and proximity for those who need continued oncology oversight.

## Figures and Tables

**Table 1:** Baseline characteristics by volume of radiation oncology follow-up during first year of survivorship.

Characteristic	Transitioned (0 Visits)	De-Intensified Follow-Up (1–2 Visits)	STD	Active Follow-Up (3+ Visits)	STD
	***N *** **= 3884**	***N *** **= 8321**		***N*** ** = 661**	
Age, in years					
Mean (SD)	74.22 (7.22)	73.25 (7.31)	**0.14**	72.63 (7.23)	**0.22**
Median (IQR)	75 (9)	74 (9)	**0.13**	73 (10)	**0.23**
Primary care access, n (%)					
Rostered	3359 (86.5%)	7290 (87.6%)	0.03	574 (86.8%)	0.01
Virtually rostered	411 (10.6%)	881 (10.6%)	0.00	68 (10.3%)	0.01
Not rostered	114 (2.9%)	150 (1.8%)	0.08	19 (2.9%)	0.00
Geographic remoteness					
Mean (SD)	0.10 (0.08)	0.09 (0.08)	**0.19**	0.11 (0.11)	0.03
Median (IQR)	0 (0)	0 (0)	**0.26**	0 (0)	0.09
Number of comorbidities					
Mean (SD)	6.45 (3.61)	6.49 (3.53)	0.01	6.59 (3.79)	0.04
Median (IQR)	6 (4)	6 (4)	0.02	6 (4)	0.03
ISUP grade, n (%)					
Group 1 (<=6)	484 (12.5%)	949 (11.4%)	0.03	42 (6.4%)	**0.21**
Group 2 (3 + 4 = 7)	1694 (43.6%)	3584 (43.1%)	0.01	251 (38.0%)	**0.12**
Group 3 (4 + 3 = 7)	833 (21.4%)	1898 (22.8%)	0.03	189 (28.6%)	**0.17**
Group 4 (8)	479 (12.3%)	1012 (12.2%)	0.01	88 (13.3%)	0.03
Group 5 (9–10)	394 (10.1%)	878 (10.6%)	0.01	91 (13.8%)	**0.11**
De novo cancer staging, n (%)					
Stage 1	379 (9.8%)	743 (8.9%)	0.03	25 (3.8%)	**0.24**
Stage 2	3202 (82.4%)	6879 (82.7%)	0.01	550 (83.2%)	0.02
Stage 3	303 (7.8%)	699 (8.4%)	0.02	86 (13.0%)	**0.17**
Treatment modality, n (%)					
External beam, prostate only, n (%)	2542 (65.4%)	4774 (57.4%)	**0.17**	382 (57.8%)	**0.16**
Brachytherapy only, n (%)	370 (9.5%)	843 (10.1%)	0.02	73 (11.0%)	0.05
External beam, prostate and pelvis, n (%)	575 (14.8%)	1134 (13.6%)	0.03	87 (13.2%)	0.05
External beam and brachytherapy, n (%)	397 (10.2%)	1570 (18.9%)	**0.25**	119 (18.0%)	**0.23**
Hormones, n (%)	1134 (29.2%)	2443 (29.4%)	0.00	218 (33.0%)	0.08
Radiation complications, n (%)					
Urologic	890 (22.9%)	1874 (22.5%)	0.01	153 (23.1%)	0.01
Rectal/anal	1091 (28.1%)	2480 (29.8%)	0.04	193 (29.2%)	0.03
Open surgery	7 (0.2%)	12 (0.1%)	0.01	***	***
Hospitalization	316 (8.1%)	424 (5.1%)	**0.12**	43 (6.5%)	0.06
Most recent PSA score, n (%)					
Undetected ^^^	829 (21.3%)	1820 (21.9%)	0.01	79 (12.0%)	**0.25**
Detected	2094 (53.9%)	4739 (57.0%)	0.06	493 (74.6%)	**0.44**
Not reported	961 (24.7%)	1762 (21.2%)	0.09	89 (13.5%)	**0.29**

Standardized differences > 0.1 considered statistically important. ^^^ Undetected PSA test defined as <=0.1 ug/L. Abbreviations: IQR = interquartile range, SD = standard deviation, STD = standardized difference, PSA = prostate-specific antigen. NOTE: table truncated for space; full version available in online-only supplemental (Table S3). *** Suppressed for privacy reasons (small cell frequency).

**Table 2:** Multivariable model estimates for radiation oncology (RO) follow-up.

Model Covariate	Adjusted *Rate* Ratio ^a^ (Model 1) Modeling Volume of RO Follow-Up	Adjusted *Risk* Ratio ^b^ (Model 2) Modeling Probability of 1 or More RO Visit During First Year of Survivorship
Age * (ref: 60 years)		
80 years	**0.83 (0.79–0.87)**	**0.95 (0.92–0.98)**
90 years	**0.69 (0.61–0.77)**	**0.87 (0.82–0.93)**
Geographic remoteness *, out of 100 (ref: 0)		
5	**0.79 (0.75–0.83)**	**0.90 (0.87–0.92)**
10	**0.70 (0.67–0.73)**	**0.87 (0.85–0.89)**
15	**0.71 (0.68–0.75)**	**0.85 (0.83–0.88)**
20	**0.74 (0.70–0.78)**	**0.85 (0.83–0.88)**
25	**0.78 (0.74–0.83)**	**0.87 (0.85–0.90)**
Primary care access (ref: rostered)		
Virtually rostered ^c^	1.04 (0.99–1.10)	0.99 (0.97–1.02)
Not rostered	**0.82 (0.71–0.94)**	**0.91 (0.85–0.98)**
Number of comorbidities (per 3-unit increase)	**1.05 (1.03–1.06)**	1.00 (1.00–1.01)
ISUP grade (ref: group 1)		
Group 2	1.06 (0.98–1.16)	1.00 (0.95–1.05)
Group 3	**1.20 (1.10–1.31)**	1.02 (0.97–1.07)
Group 4	**1.25 (1.14–1.38)**	1.00 (0.95–1.06)
Group 5	**1.34 (1.21–1.48)**	1.02 (0.96–1.07)
Staging (ref: I)		
II	**1.15 (1.04–1.26)**	1.03 (0.97–1.09)
III	**1.29 (1.15–1.44)**	1.05 (0.99–1.12)
PSA score (ref: undetectable)		
Detectable	**1.21 (1.16–1.26)**	**1.03 (1.00–1.05)**
Not reported	1.02 (0.97–1.06)	0.98 (0.95–1.01)
Radiation modality (ref: external beam, prostate)		
Brachytherapy	**1.16 (1.08–1.23)**	**1.04 (1.01–1.08)**
External beam, prostate and pelvis	0.98 (0.93–1.03)	0.99 (0.96–1.02)
External beam and brachytherapy	**1.11 (1.07–1.16)**	**1.10 (1.07–1.13)**
Hormones (ref: none)	**1.05 (1.00–1.10)**	**1.04 (1.01–1.07)**
Radiotherapy complications (ref: none)		
Urologic	**1.20 (1.16–1.24)**	1.00 (0.98–1.03)
Rectal/anal	**1.10 (1.06–1.13)**	**1.03 (1.01–1.05)**
Open surgery	**1.25 (1.02–1.54)**	1.03 (0.82–1.31)
Hospitalization	**0.94 (0.89–1.00)**	**0.91 (0.87–0.95)**
Follow-up visits to urology ^#^ (per 1-visit increase)	**0.88 (0.87–0.90)**	N/A
Follow-up visits to GP/family medicine ^#^ (per 1-visit increase)	**0.95 (0.95–0.96)**	N/A

^a^ Estimated from an Anderson-Gill counting process model (recurrent event framework). ^b^ Estimated from a log-binomial model (probability of having 1 or more RO visits). ^c^ Virtual rostering denotes patients seeking primary care without being formally rostered to primary care physician. * Continuous variable adjusted using restricted cubic splines with five knots. ^#^ Adjusted as time-varying covariate (not possible with log-binomial model). Abbreviations: N/A = not applicable, REF = reference group. NOTE: table truncated for space; full version available in online-only supplemental (Table S4).

## Data Availability

The dataset from this study is held securely in coded form at ICES. While legal data sharing agreements between ICES and data providers (e.g., healthcare organizations and government) prohibit ICES from making the dataset publicly available, access may be granted to those who meet pre-specified criteria for confidential access, available at www.ices.on.ca/das (email: das@ices.on.ca). The underlying analytic code is available from authors upon request, understanding that the computer programs may rely upon coding templates or macros that are unique to ICES and are, therefore, either inaccessible or may require modification.

## Supplementary Materials

The following supporting information can be downloaded at: https://www.mdpi.com/article/10.3390/curroncol33010049/s1, Figure S1: Cohort exclusions; Figure S2: Kaplan-Meier curve for all-cause mortality; Table S1: Description of data sources; Table S2: Variable definitions; Table S3: Baseline characteristics by volume of radiation oncology follow-up during first year of survivorship (full version); Table S4: Multivariable model estimates for radiation oncology (RO) follow-up (full version).
